# Conjugation of Gentamicin to Polyamidoamine Dendrimers Improved Anti-bacterial Properties against *Pseudomonas aeruginosa*

**DOI:** 10.34172/apb.2021.076

**Published:** 2020-10-14

**Authors:** Hamed Sheykhloo, Morteza Milani, Farhood Najafi, Farhad Bani, Amir Zarebkohan

**Affiliations:** ^1^Biotechnology Department, Rabe Rashidi University, Tabriz, Iran.; ^2^Department of Medical Nanotechnology, Faculty of Advanced Medical Sciences, Tabriz University of Medical Sciences, Tabriz, Iran.; ^3^Department of Resin and Additives, Institute for Color Science and Technology, Tehran, Iran.; ^4^Drug Applied Research Center, Tabriz University of Medical Sciences, Tabriz, Iran.

**Keywords:** Gentamicin, PAMAM dendrimers, Pseudomonas aeruginosa, Drug delivery, Anti-bacterial capacity

## Abstract

***Purpose:*** This study aimed to design gentamicin-conjugated poly (amidoamine) (PAMAM) dendrimers to increase the therapeutic efficiency of gentamicin against *Pseudomonas aeruginosa*.

***Methods:*** Gentamicin-presenting dendrimers were synthesized using MAL-PEG_3400_-NHS as a redox-sensitive linker to attach gentamicin to the surface of G4 PAMAM dendrimers. The gentamicin molecules were thiolated by using Traut reagent. Then, the functionalized gentamicin molecules were attached to PEGylated PAMAM dendrimers through simple and high selectively maleimide (MAL)-thiol reaction. The structure of gentamicin-conjugated PAMAM dendrimers was characterized and confirmed using *nuclear magnetic resonance* (*NMR*), dynamic light scattering (DLS), zeta potential analysis, and *transmission electron microscopy* (TEM) imaging. The antibacterial properties of the synthesized complex were examined on *P. aeruginosa* and compared to gentamycin alone.

***Results:*** NMR, DLS, zeta potential analysis, and TEM imaging revealed the successful conjugation of gentamicin to PAMAM dendrimers. Data showed the appropriate physicochemical properties of the synthesized nanoparticles. We found a 3-fold increase in the antibacterial properties of gentamicin conjugated to the surface of PAMAM dendrimers compared to non-conjugated gentamicin. Based on data, the anti-biofilm effects of PAMAM-Gentamicin dendrimers increased at least 13 times more than the gentamicin in normal conditions.

***Conclusion:*** Data confirmed that PAMAM dendrimer harboring gentamicin could be touted as a novel smart drug delivery system in infectious conditions.

## Introduction

Bacterial infections remain major health challenges worldwide and antimicrobial drug resistance is considered as one of the most unsettled medical problems.^1,2^ One of the most important mechanisms used by antibiotic-resistant bacteria such as *Pseudomonas aeruginosa*, a gram-negative bacterium, is the development of biofilm around infectious agent.^3^ Different multidrug-resistance strains of this pathogen are linked with life-threatening diseases like chronic obstructive pulmonary disease, cystic fibrosis, and also *P. aeruginosa* is the foremost reason of death in patients with extensive burns.^4,5^ Gentamicin belongs to the aminoglycoside class of antibiotics and is used widely to treat several types of bacterial infections.^6^ Gentamicin is uptake in three distinct steps by bacterial cells as follows; an initial binding to the bacterial membrane, and chased by two energy-dependent stages (called EDP-I and EDP-II). The uptake of gentamicin in EDP-I occurs slowly which surveyed by energy-dependent accumulation of the gentamicin inside bacteria in the next step namely EDP-II.^6,7^ The most accepted concept for the bactericidal function of gentamicin proposes that a small amount of gentamicin enters inside the bacterium in the phase I (EDP-II) and lasts until the second step phase II (EDP-II). Thereafter, this antibiotic could bind to ribosomes, resulted in disrupted multiple proteins by misreading of mRNAs.^6^ As a result, membrane integrity is destroyed. This phenomenon contributes to the formation of pores, an increase of osmotic pressure, ion imbalance, and irreversibly saturated ribosomes, resulting in bacterial loss.^6^ Drug resistance mechanisms are compensatory mechanisms to resist different antibiotics. Under these circumstances, the expression of genes associated with the construction of multiple proteins including enzymes for drug elimination (β-lactams), drug resistance pumps (efflux pumps), and membrane integrity is increased to prevent bactericidal effects.^6,8^ Unfortunately, numerous studies have shown that *P. aeruginosa* is resistant to a variety of aminoglycosides, since 1973. Moreover, the high doses of gentamicin predispose patients to nephrotoxicity and ototoxicity.^6,9^

Despite the urgent need to find new drugs to overcome drug resistance, the development of novel classes of antibiotic agents with high antibacterial activity and wide therapeutic window are crucial. In recent years, nano-formulated drug delivery systems have been settled to increase the therapeutic efficiency against bacterial infections, considering new and powerful strategies.^10,11^ The development of stimuli-responsive nano-carriers can be used to combat bacteria upon reaching bacterial microenvironment.^12^ Depending on the pathological conditions of infections, stimuli-responsive nanoparticles can respond to the specific pH, enzymes, or chemicals.^13^ To date, different modalities have been applied for the conjugation of smart chemical to the target molecules. Maleimide (MAL) derivatives are extensively used for coupling with thiol moieties in the structure of proteins, peptides, and drugs.^14^ Unfortunately, nearly all MAL-thiol adducts linkage is recognized unstable under physiological conditions. In the existence of free thiol groups provided by different amino acids such as, cysteine serum albumin, and glutathione, the linkage is reduced via a retro-Michael or thiol exchange reaction.^15^ Based on the strength of exogenous thiol nucleophiles (R-SH), the attached drugs are reduced and released from redox responsive drug delivery system.^16,17^ Poly (amidoamine) (PAMAM) dendrimers are hyper-branched, nano-sized globular polymers with tailorable surface functionality which are a great tool for numerous biomedical applications such as drug and gene delivery systems.^18^ In this regard, drug molecules can be either encapsulated or covalently attached to the dendrimers by engaging functional groups on the surface. In support of this notion, inhibition of bacterial growth has been shown in some studies by PAMAM dendrimers with different surface functionality.^19,20^

To the best of our knowledge, we did not find similar reports available on the attachment of specific antibiotics to the dendrimer surface using MAL-thiol linkage to develop a smart nano-drug delivery system against bacterial infections. Here, we evaluated the possible cytotoxicity and *in vitro* antibacterial capacity of gentamicin against *P. aeruginosa* after being-conjugated to the G4 PAMAM dendrimer surface. The main goal of this experiment was to improve the therapeutic outcome of gentamicin against drug-resistant bacteria by developing a gentamicin modified PAMAM dendrimer system using MAL-PEG_3400_-NHS redox-sensitive linker.

## Materials and Methods

### Materials

G4 PAMAM dendrimer (MW: 14215 Da) with an ethylenediamine core and methyl acrylate was synthesized by modified Michael-amidation method.^21^ Briefly, this method consists of alkylation with methyl acrylate and subsequent amidation with ethylenediamine.^18,21^ Bifunctional PEG (NHS-PEG-MAL, MW: 3400) was purchased from Nanocs Inc. (Boston, USA). Gentamicin sulfate was a gift from Zahravi Pharmaceutical Company (Tabriz, Iran), Amicon Ultra-15 Centrifugal Filter (15 kDa), and 2-iminothiolane hydrochloride (Traut reagent) were purchased from Sigma-Aldrich.

### Synthesis and characterization of PAMAM-gentamicin-conjugate nanoparticles

#### Conjugation of bifunctional PEGs to PAMAM (PAMAM-PEG)

To initiate the reaction of PAMAM primary amine groups to NHS groups of bifunctional PEG, the PAMAM dendrimers were incubated with PEGs in a molar ratio of 1:1 (NH2 of dendrimer: PEG, Mol/Mol) in PBS (pH= 8) for 2 hours at room temperature according to our previously published data.^21^ To exclude unreacted PEGs, Amicon Ultra Centrifugal Filters with cut off 15 kDa were used.

### Gentamicin thiolation

To thiolate gentamicin, gentamicin dissolved in PBS and reacted with 2-iminothiolane hydrochloride (Traut’s reagent) in a 1:2 molar ratio (pH 8.3) for 24 hours.

### Conjugation of thiolated gentamicin with PAMAM (PAMAM-PEG-gentamicin)

The PAMAM-PEG conjugates were incubated with thiolated gentamicin in a molar ratio of 1:1 (NH_2_ of dendrimer: gentamicin, Mol/Mol) in PBS (pH=7.0) at room temperature for 24 hours. Terminal MAL groups of PAMAM–PEG can interact exclusively with thiol groups of gentamicin. The PAMAM-PEG-Gentamicin conjugates were purified by dialysis (MW cut-off 10 kDa).

### Characterization of synthesized nanoparticles

The physicochemical characteristics of gentamicin-conjugated PAMAM dendrimers were evaluated by *nuclear magnetic resonance* (*NMR*), dynamic light scattering (DLS), and *transmission electron microscopy (*TEM) imaging. To perform the NMR analysis, PAMAM dendrimers, PAMAM–PEG, and PAMAM–PEG–Gentamicin conjugates were initially lyophilized, dissolved in heavy water (D_2_O), and analyzed by using Bruker 400 MHz Avance II + NMR spectrometer system. Features such as size and zeta potential values were studied using DLS and zeta plus analyzer (Malvern Zetasizer Nano ZS, UK). The drug loading capacity (DL) was calculated using the following formula:

DL% = Weight of drug in dendrimer/Weight of dendrimer containing drug ×100

### Measuring minimum inhibitory concentration (MIC)

In this study, the standard strain of *P. aeruginosa* (ATCC^®^ 27853) was used to assess the antimicrobial effect of PAMAM, gentamicin, and PAMAM-PEG-Gentamicin conjugates. The MIC value was calculated using a micro-broth dilution method. For this purpose, the strain was cultured in nutrient broth medium and incubated at 35˚C for 24 hours. Then, suspensions cultures were prepared in sterile normal saline to a density equal to No. 0.5 McFarland turbidity standard according to the CLSI recommendation.^22^ The serial dilutions were prepared in 8 test tubes containing nutrient broth medium. The bacterial suspension was diluted 1:100 using sterile Nutrient broth, transferred in each tube, and incubated at 35°C for 24 hours. After incubation at 35°C, inhibition of growth bacterial isolates was determined. The MIC value was defined as the lowest concentration that inhibited detectable growth.

### Time-kill study assay

The time-kill study of PAMAM-PEG-gentamicin nanoparticles was carried out based on the procedure described by Tsuji et al.^23^ MIC and 2MIC levels of PAMAM-PEG-Gentamicin nanoparticles were used. Briefly, tubes containing 2 mL of nutrient broth medium with a concentration of MIC and 2MIC were prepared and then were incubated at 35°C for 24 hours. At intervals of 0, 3, 6, 9, and 24 hours, the contents of the tubes were gently mixed and the number of bacteria in each tube was estimated using the pour-plate method. Dilutions of 1: 10, 1: 100, and 1: 1000 were prepared, and the pour-plate procedure was performed. One tube without antibiotics was used as a positive control.

### Calculation of minimal biofilm eradication concentration (MBEC)

The ability of *P. aeruginosa* strain to form biofilm was evaluated after being incubated with PAMAM, gentamicin, and PAMAM-PEG-Gentamicin conjugates.^24^ Serial dilutions of both forms of gentamicin were prepared and poured in each well of microtiter plates containing culture media plus 0.5 McFarland of the bacterial suspension. The group with free gentamicin was considered as positive control and data compared with the gentamicin-free group (negative control), respectively. The cut off optical density for biofilm formation was considered as follows; lack of biofilm (-), weak (+), moderate (++), and strong formation (+++). To optimize results and provide reliable analysis of data, three sets of experiments were conducted.

## Results and Discussion

### Synthesis and characterization of nanoparticles

**Figure 1:**
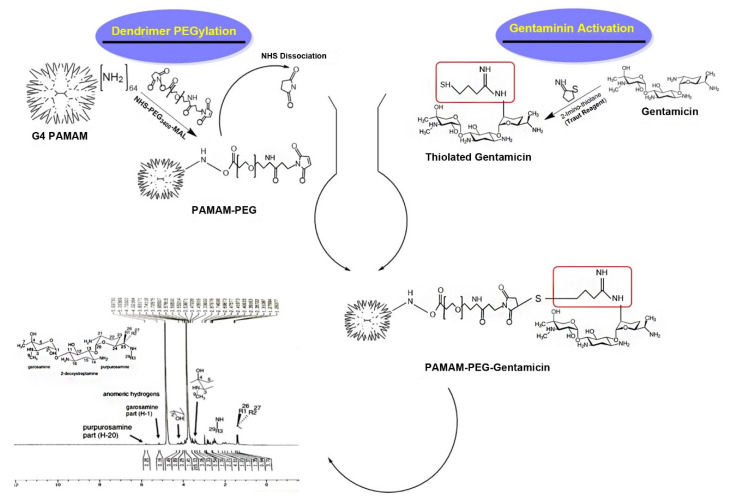
The schematic of the synthesis process of PAMAM-PEG_3400_-gentamicin nanoparticles.

According to our data, G4 PAMAM dendrimer has theoretically 64 numbers of peripheral branches each terminated with a primary amine susceptible to covalent conjugation with a variety of small molecules. The development of gentamicin-modified G4 PAMAM dendrimer occurs via gentamicin and dendrimer amine groups. Gentamicin conjugated G4 PAMAM dendrimers were produced in a three-step chemical reaction (Figure 1). The first step involves the conjugation of hetero cross-linker MAL-PEG_3400_-NHS to PAMAM dendrimers amine groups, in the second step, gentamicin molecules were thiolated by Traut reagent, and finally, the thiolated gentamicin molecules were conjugated with PEGylated PAMAM dendrimers. The main rationality behind our design was the possibility of gentamicin release by the elimination of MAL-thiol adducts linkage in condition exposed to Pseudomonas aeruginosa and intracellular thiol nucleophiles such as glutathione content and/or thioredoxin reductase enzyme.^25^

**Figure 2:**
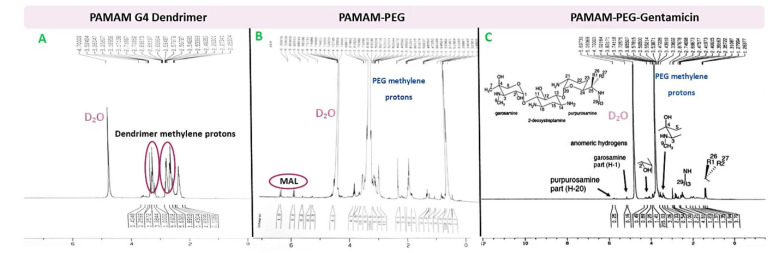
^1^H NMR spectrum of A) G4 PAMAM dendrimer, B) PAMAM-PEG and C) PAMAM-PEG-Gentamicin.

In the first step, PAMAM-NH2 was reacted with the N-hydroxysuccinimide (NHS) ester of NHS-PEG_3400_-MAL (Figure 1). The PEGylated PAMAM dendrimers were confirmed by proton NMR analysis (Figure 2A and 2B). Chemical shifts of methylene protons belong to the PEG (3.6 ppm, 4H), and MAL group methylene protons appeared after the conjugation. The integration of characteristic peak for PEG (methylene protons 3.6 ppm) was obtained from NMR analysis and compared to the PAMAM values (2.49-2.59 ppm: CH2-CH2CONH), suggesting that 18 molecules of PEG were attached to the PAMAM. Gentamicin molecules were thiolated by using Traut reagent. This reagent has been used widely by researchers for the attachment of antibodies to drug molecules or nanoparticles.^26-28^ Thiolated gentamicin was then reacted with PEGylated PAMAM to MAL-thiol coupling (Figure 1). The conjugation of the gentamicin on the PAMAM surface was confirmed by proton NMR, showing characteristic peaks for the gentamicin shown in Figure 2C. Complete removal of the MAL methylene protons peak (at 6.1 and 6.8 ppm) showed that 18 molecules of gentamicin were attached to the surface of the PEGylated PAMAM dendrimers.

**Figure 3:**
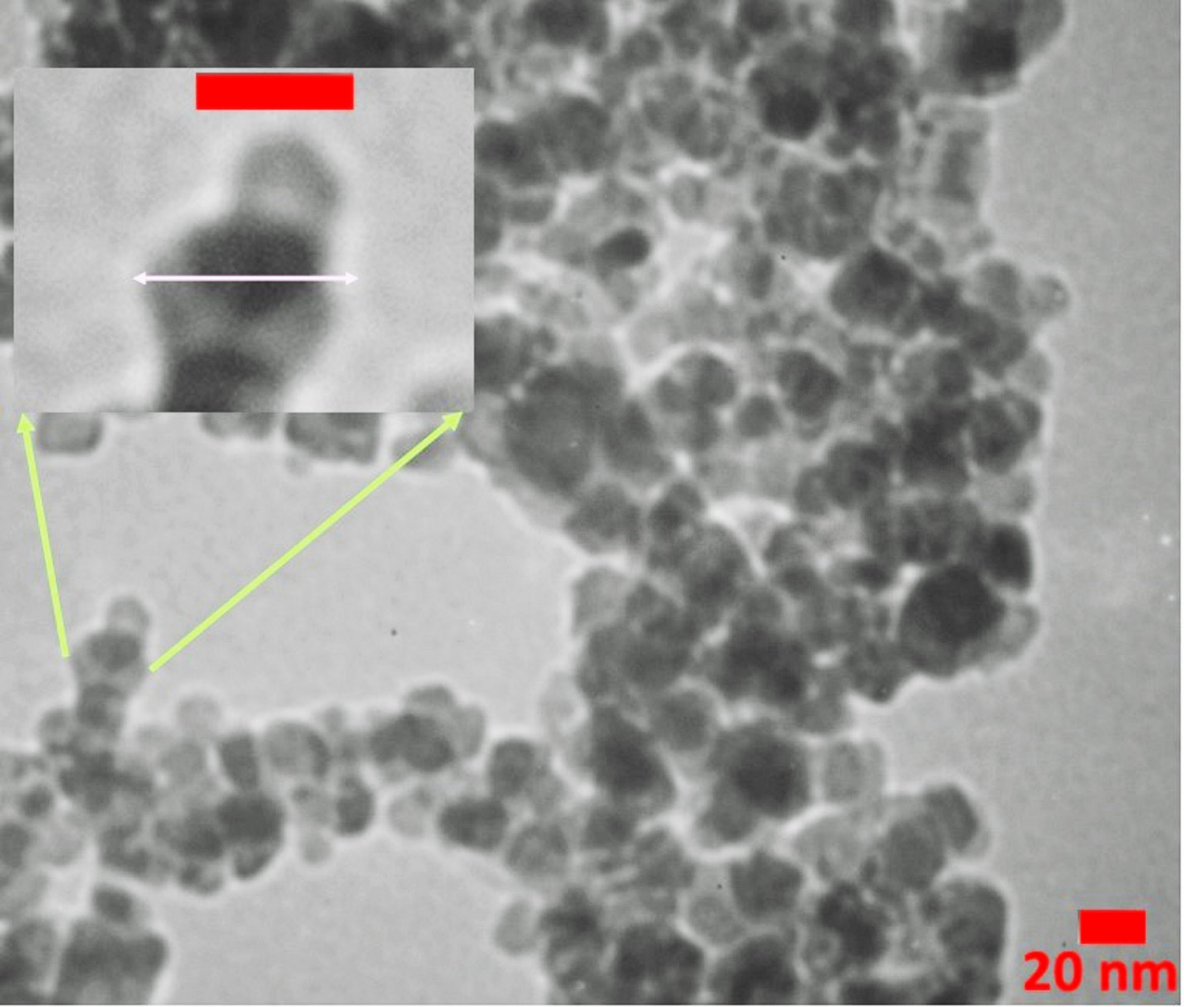
TEM image of gentamicin conjugated PAMAM dendrimers.

The drug loading capacity was about 59% by weight, which is high amount for a polymer-drug conjugate. Also, the conjugated form of gentamicin was soluble in PBS buffer (pH 7.4) compared to the free form. The conjugation of gentamicin with PAMAM was also confirmed by DLS analysis (Table 1). The PAMAM G4 dendrimers presented a positive zeta potential of 18.5 ± 3.1 mV in deionized water due to cationic amine groups on the surface. After conjugation of thiolated gentamicin with the dendrimer surface, the zeta potential of PAMAM-PEG-Gentamicin nanoparticles was decreased to -8.8 ± 3.9 mV. This property is due to the reduction of PAMAM amine groups by PEG coupling and the existence of hydroxyl and remained free thiol groups in the structure of conjugated gentamicin. Also, the hydrodynamic diameter of PAMAM dendrimer was increased from 4.4 nm to 51.2 ± 6.6 nm after the formation of gentamicin conjugates, indicating successful gentamicin link to PAMAM dendrimers. TEM images (Figure 3) showed that the PAMAM-PEG-Gentamicin nanoparticles had a uniform spherical shape and ~28 nm size which is consistent with theoretical predictions for PEGylated G4 PAMAM dendrimers.

**Table 1:** Hydrodynamic diameter and Zeta potential of nanoparticles

**Nanoparticle name**	**ζ Potential (mV)**	**Size by number (nm)**	**Polydispersity index (PDI)**
PAMAM dendrimer	18.5±3.1	4.4±0.8	0.2
PAMAM-PEG	9.4±1.2	45.6±5.3	0.3
PAMAM-PEG-gentamicin	-8.8±3.9	51.23±6.6	0.2

PDI, Polydispersity index

### Determination of MIC

**Figure 4:**
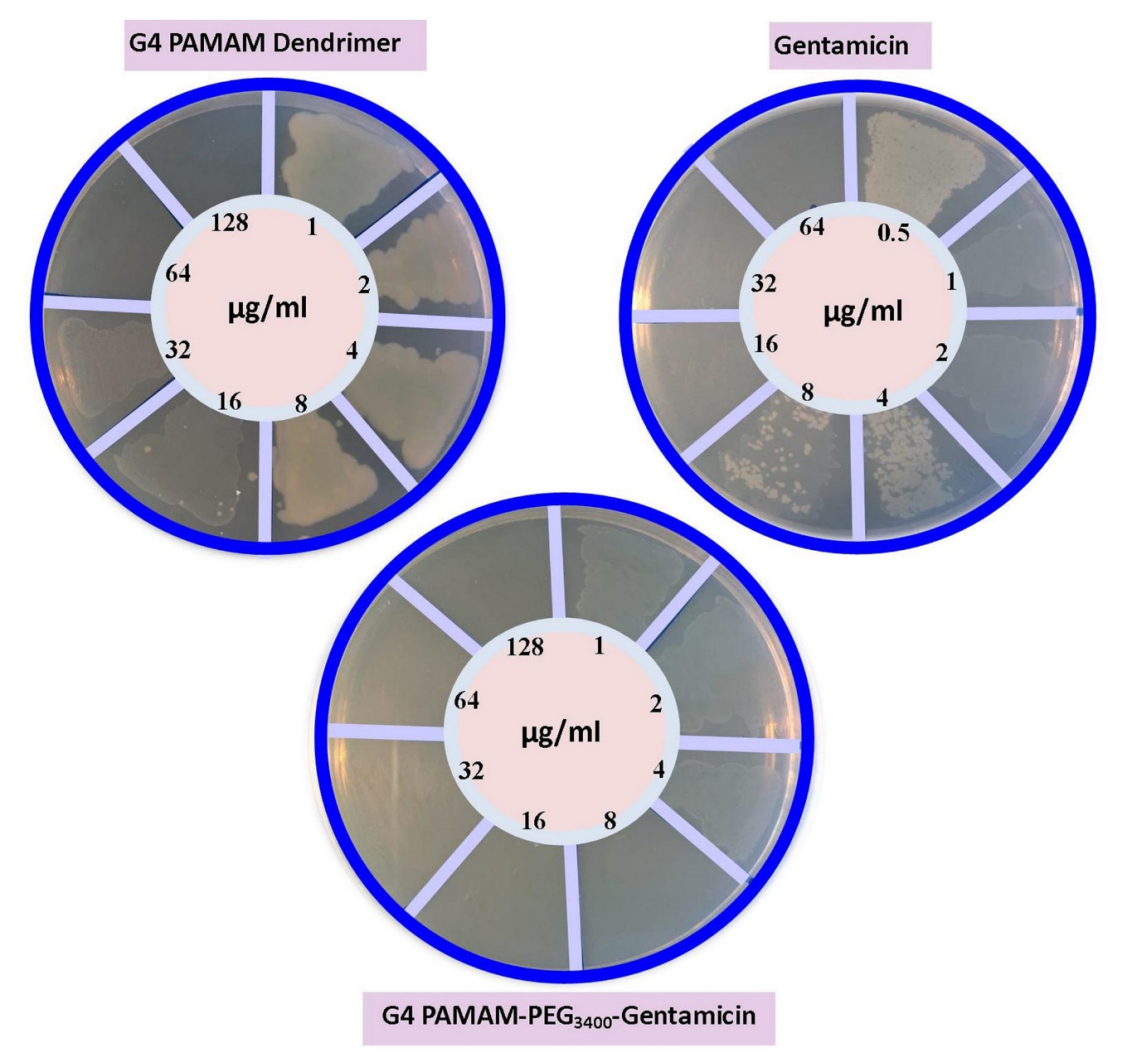
The evaluation of compounds minimum inhibitory concentration (MIC).

Data showed statistically significant differences in the MIC values of groups treated with free and conjugated forms of gentamicin. In this study, the MIC of the free and conjugated forms of gentamicin against *P. aeruginosa* was 16 and 4.7 μg/mL, respectively. The MIC of PAMAM was 64 μg/mL (Figure 4). According to our data, the MIC of gentamicin was within the range previously specified by the Clinical & Laboratory Standards Institute. Interestingly, the binding of gentamicin to dendrimer significantly reduced the MIC.

**Figure 5:**
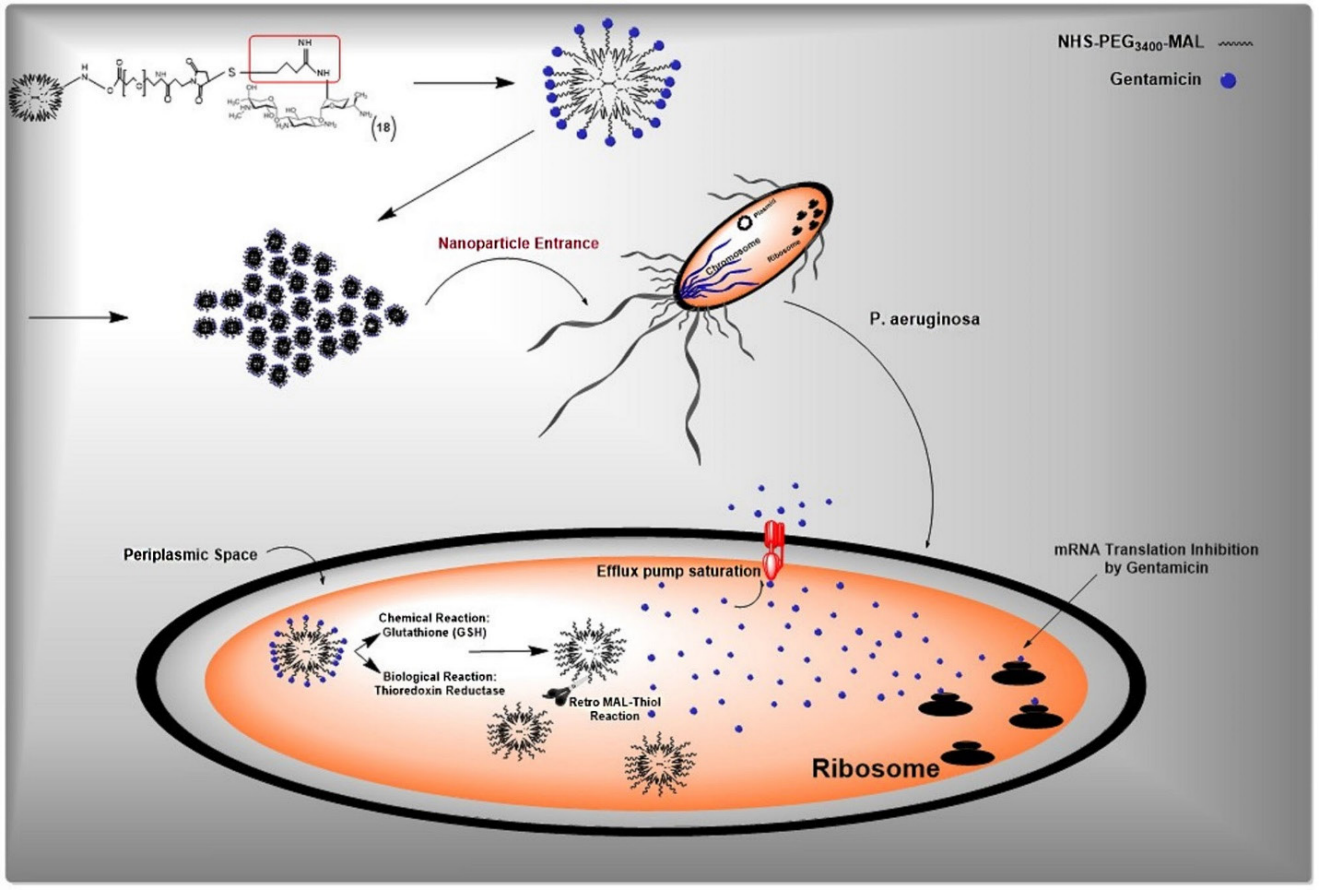
Redox-responsive mechanism of drug release from developed nanoparticles.

The antibacterial mechanisms that have been proposed for such nanoparticles so far include the destruction of bacterial cell walls or enhanced drug delivery into the bacterial cell.^29^ Since the synthesized nanoparticles have a negative charge, the possibility of bacterial cell membrane demolition is ruled out and enhanced drug delivery should be the main mechanism. Considering the mechanism of action of gentamicin, and spatial point of view, it seems that gentamicin-modified dendrimers cannot attach to the ribosomal 30s subunit properly.^30-32^ Compared to free gentamicin and dendrimer alone, the prominent antibacterial effect of gentamicin-conjugated PAMAM dendrimers is possibly associated with the intracellular release of gentamicin occurred chemically in response to glutathione content and enzymatically by the activity of thioredoxin reductase through the increase of retro-reaction of MAL-thiol adducts (Figure 5).^25,33^ This phenomenon has previously been reported in a wide range of studies.^15,17,34,35^ We hypothesized that the sudden increase of gentamicin concentration inside the bacteria saturates the function of MDR pumps. Consequently, efflux of gentamicin as an important antibacterial resistance mechanism abolished, and therefore the bacterial cells eradicated without an effective struggle.

### Time-kill study

**Figure 6:**
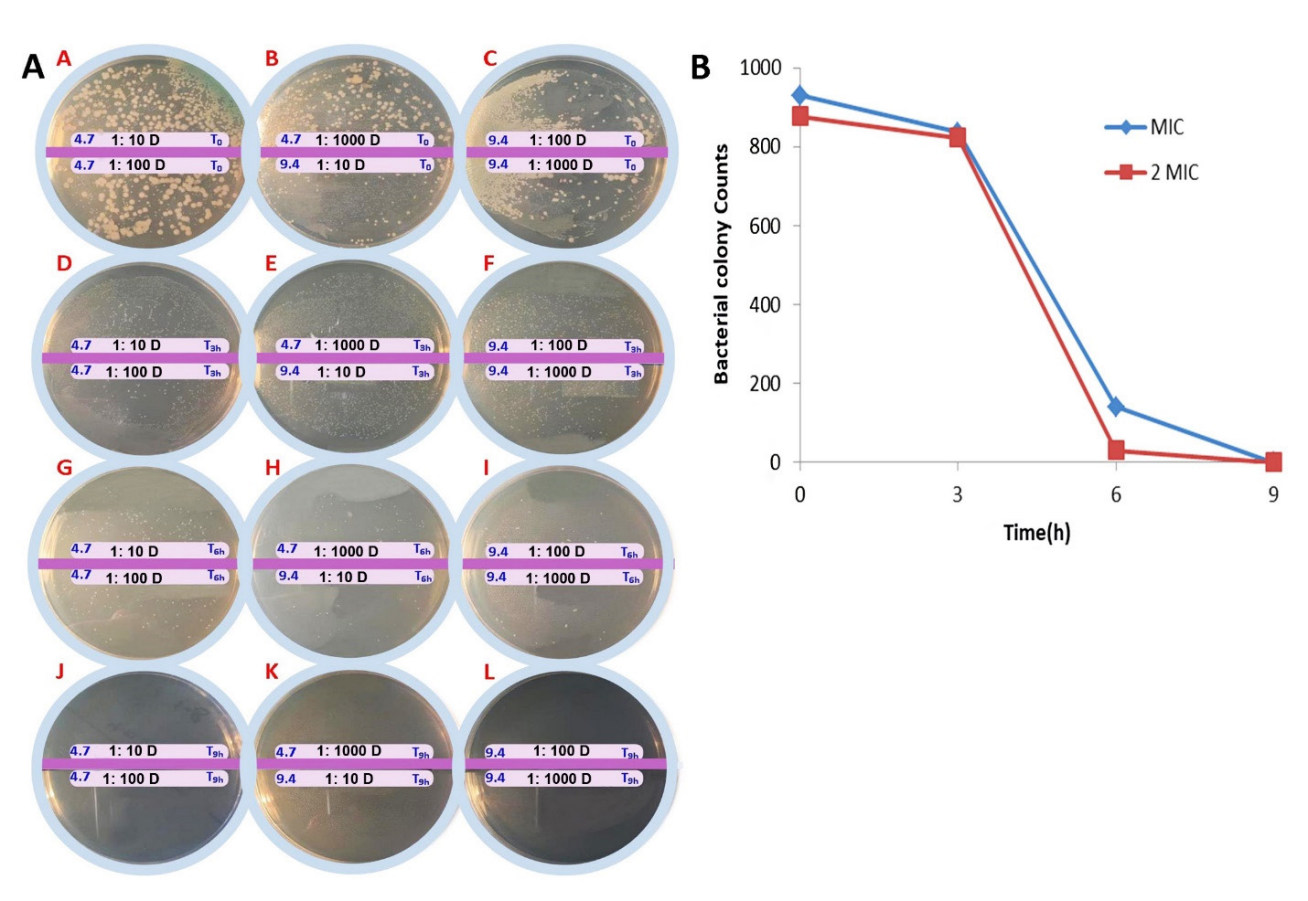
Time kill study assay. (A) The cultured plate at time 0 (A-C), 3 h (D-F), 6 h (G-I), and 9 h (J-L) after incubation with MIC (~4.7 µg/mL) and 2MIC (~9.4 µg/mL) of PAMAM-PEG-Gentamicin. The dilutions of 1:10, 1:100 and 1:1000 were prepared and then pour-plate was performed. (B) Time-kill study curves with P. aeruginosa. The concentrations of gentamicin used represent the respective MIC and 2MIC.

Time-kill study test showed that PAMAM-PEG-Gentamicin nanoparticles reduced the number count of living bacteria at 1MIC and 2MIC concentrations after 3 and 6 hours in which no colonies were found 9 hours after incubation (Figure 6). The 3-hour incubation of *Pseudomonas aeruginosa* with 1MIC reduced viable bacteria from 7×10^4^ to 7×10^3^ CFU/mL and these values reached 1×10^2^ CFU/mL after 6 hours. As expected, treatment of bacteria with 2MICs diminished viable cell count from 7×10^4^ at time point 0 to 5×10^2^ and 2×10^1^ CFU/mL after 3 and 6 hours respectively after being exposed to PAMAM-PEG-Gentamicin nanoparticles. No viable colonies were showed at both 1MIC and 2MIC after 9 hours. These features showed the dose-dependent activity of PAMAM-PEG-Gentamicin nanoparticles on *P. aeruginosa.*

### Determination of MBEC

Biofilm is a complex structure which bacteria fenced by using the matrix of extracellular polymer substances which released by the microbes themselves.^36^ It has been shown that the high resistance and rigid structure of biofilm prevent the permeation of antimicrobial drugs, playing a vital role in the failure of clinical medication for infections associated with *P. aeruginosa*. Therefore, researchers emphasize that biofilm eradication is an effective strategy in controlling microbial infections.^37^ In current study, we measured the MBEC in the presence of free gentamicin and PAMAM-PEG-Gentamicin nanoparticles. Our result showed that biofilm formation capacity was reduced from 4.7 up to 0.3 µg/mL in the groups treated with PAMAM-PEG-Gentamicin nanoparticles (Table 2). However, we noted that the formation of biofilm was completely inhibited in the gentamicin group at the concentrations between 16 and 64 µg/mL. It seems that PAMAM could decrease the formation of biofilm in 16 µg/mL more effectively than 64 µg/mL (Table 2). Overall, we showed that the conjugation of gentamicin with PAMAM can significantly inhibit biofilm formation in *P. aeruginosa* at lower concentrations than that of free gentamicin.

**Table 2:** Minimum biofilm eradication concentration of free gentamicin (G) and conjugated gentamicin form (PAMAM-PEG-Gentamicin) on *Pseudomonas aeruginosa*

**Anti-bacterial agents**	**Minimum Biofilm Eradication Concentration Assay (MBEC µg/mL)**							
PAMAM	0.5	1	2	4	8	16	32	64
	+++	+++	+++	+++	+++	+++	+	-
Gentamicin	0.12	0.25	0.5	1	2	4	8	16
	+	+	+	+	+	--	--	--
PAMAM-PEG-gentamicin	0.03	0.07	0.15	0.3	0.6	1.2	2.3	4.7
	+	+	+	--	--	--	--	--

- (no biofilm formation), + (weakly), ++ (moderate), +++ (strong) biofilm formation.

Selective antibiofilm properties of PAMAM-PEG-Gentamicin nanoparticles are associated with the appropriate size of nanoparticles. It has been shown that the decrease of nanoparticle size increased the penetration capacity through tight mucosal and fibroid structural biofilms.^38^

The rationale behind the development of redox-responsive dendrimers is to use disulfide bonds for conjugation in the structure of the nano-based drug delivery system.^39,40^ Some studies were conducted to develop redox-sensitive nano-drug delivery systems. For instance, the *in situ* biodegradable hydrogels have been developed using generation 4 poly (amidoamine) [G4-(NH2)_64_] and thiol terminated 8-arm polyethylene glycol (PEG) as an amoxicillin control released platform.^40^

Yan et al designed another smart nanostructure by using dendrimers containing hydrophobic di-sulfide units, which are assembled and de-assembled in the presence of GSH.^41^ In another work, the organometallic dendritic scaffold has been used to the eradication of vancomycin-resistant *Enterococcus faecium* and methicillin-resistant *Staphylococcus aureus* by the destruction of the cell wall and the generation of oxidative stress.^42^ Because of similar effects on healthy cells, these positively charged nano-structures efficiently destroy the lipid membrane, which limits their routine application. Wang and co-workers created a PAMAM dendrimer/gold nanoparticles with redox-responsive capability for delivering thiolated anticancer agents such as 6-mercaptopurine and captopril, via the foundation of Au–S bond.^43^ The results showed that drugs were released from this structure in the “OFF-ON” manner after exposure to the high amount of intracellular GSH similar to our developed nanoparticle. Additionally, Du et al. designed a redox-sensitive Janus dendrimer for the delivery of siRNA. Because of disulfide linkages in the structure of the dendrimers, siRNA was released in response to the cell’s redox system.^44^ Compared to these studies, our concept in the design of nanoparticles has some advantages, including simple synthesis protocol without the necessity to use additional disulfide linkers which can affect the nanoparticle integrity and limit their application to the clinical setting.

## Conclusion

In the present study, we used successfully the MAL-thiol reaction to develop a smart drug delivery system based on gentamicin-PAMAM dendrimers to increase the intracellular delivery of gentamicin in *P. aeruginosa*.It seems that the intracellular release of gentamicin occurs chemically and enzymatically by the increase of the kinetics of retro-reaction of MAL-thiol adducts. The efflux of gentamicin could be abolished by a sudden increase of inside gentamicin concentration leading to saturation of MDR. The synthesized nanoparticles can be also used to inhibit antibiotic-resistant mechanisms such as biofilm formation at lower concentrations compared to the free drug. The current strategy seems to be appropriate to treat infections caused by *P. aeruginosa*.

## Ethical Issues

All authors confirmed that this work has not been previously published elsewhere and is not currently being considered for publication elsewhere. Also, the authors confirm that all of the experiments adjusted to the ethical committee of Tabriz University of Medical Sciences.

## Conflict of Interest

The authors declare that there is no conflict of interest.

## Acknowledgments

This work is a part of a MSc. the thesis of Hamed Sheykhloo in Microbial Biotechnology at Rabe Rashidi University, Tabriz, Iran. This study was financially supported by young researchers welcome grant [NO: 58859] from the Faculty of Advanced Medical Science, Tabriz University of Medical Sciences, Tabriz, Iran.
